# BFLAFD: blockchain-enabled federated learning framework for adaptive fire detection in IIoT networks

**DOI:** 10.1038/s41598-026-51138-1

**Published:** 2026-05-05

**Authors:** Jayameena Desikan, Sushil Kumar Singh, A. Jayanthiladevi, Himanshu Gupta

**Affiliations:** 1 Department of Computer Engineering, Marwadi University, Rajkot, Gujarat India; 2 Department of Computer Science and Engineering, Adichunchangiri University, Karanataka, India; 3 Manipal Institute of Technology, Manipal Academy of Higher Education, Manipal, India

**Keywords:** Industrial Internet of Things (IIoT), Federated Learning (FL), Blockchain, Personalized Federated Learning (PFL), Fire Detection, Engineering, Mathematics and computing

## Abstract

The Industrial Internet of Things (IIoT) is a network of interconnected sensors, devices, and control systems in the oil and gas sectors that has been developed to make the industries automated and continuously monitored. However, there are challenges in fire detection in such environments, including the unreliable nature of the sensor data, privacy issues, communications delays, and the lack of a generalized model across locations in a distributed solution. To overcome the above problems, BFLAFD, Blockchain-assisted Federated Learning framework for Adaptive Fire Detection is introduced. Unlike centralized methods, BFLAFD makes use of Federated Learning (FL), where local edge servers train models directly on-device in which data confidentiality is upheld and less data is transmitted. Hierarchical aggregation process is effective in maximizing global performance, in addition to being sensitive to sensor drift and device heterogeneity. To make sure of trust and resilience, BFLAFD combines the permissioned blockchain with smart contracts providing access control, transparency of logs and no tampering of models or insider manipulation. Furthermore, Personalized Federated Learning (PFL) makes it possible to create a customized fire detection model, effectively enhancing the accuracy in varying conditions. Experimental evaluations have shown that BFLAFD has 98.2% detection accuracy, false alarm rate of 2.7%, and a 100–150 ms inference latency, and blockchain validation time of 1–2 s. In addition, the cost of communication was reduced by 82.3% compared to centralized training. Overall, BFLAFD offers critical IIoT environments fast, accurate, and secure fire detection solutions.

## Introduction

The adoption of Industrial Internet of Things (IIoT) technologies provides industrial sectors with multiple advantages, including enhanced automation, predictive maintenance, and continuous monitoring. Smart systems that have sensors and intelligent devices with edge computing enable continuous data sharing and automatic decision-making. Safety concerns remain a top concern in critical high-risk domains such as oil and gas IIoT networks. Fire hazards endanger human lives and simultaneously damage critical infrastructure, resulting in substantial economic losses in these IIoT environments. The traditional centralized fire detection systems prove ineffective in managing the dynamic and distributed nature of IIoT networks. Real-time operation of massive heterogeneous sensor data, while maintaining privacy and security, remains a primary obstacle, which also hinders the implementation of industrial sites customized adaptations. Industrial operations moving towards autonomous systems need fire detection solutions that combine accuracy with operational scalability, along with secure and privacy-preserving solutions. In addition to these challenges, sensor data in oil and gas industrial environments often has multiple issues, which are uncertainty, noise, and inconsistencies due to harsh operational and environmental conditions. Existing centralized models also face difficulties in handling the diversity of sensor types. They are also affected by the deployment conditions and rapidly changing environments across different types of oil and gas industrial sites. Also, as more systems become connected, it is expected that there is a higher risk of attacks from inside the network, tampering with models, and changes being made to the data. To tackle these risks, a Zero Trust security approach is proposed.

In this, each and every device and data exchanges need to be authenticated and verified. Real-time fire detection capability is also achieved in the edge layer and communication delays are kept to a minimum. Fire detection models need to be able to continually learn and adapt in order to keep themselves accurate as environmental and operational conditions change on a daily basis. Blockchain technology when used in conjunction with personalized federated learning (PFL) and hierarchical federated learning (HFL)) and smart contracts, offers a decentralized method to secure data, automate trust validation, ensure model integrity and manage permissions across the distributed edge nodes^1^. PFL will guarantee that each edge device is modelled to enhance the accuracy of detection. HFL enhances the scalability by aggregating model updates in geographically distributed regions. Smart contracts are part of automation in verifying model updates and firing events reports. It guarantees tamper resistant records and access control. A smart fire detection system should be decentralized, flexible, secure to respect people’s privacy, and resilient to environmental variations and security threats. In accordance with the goals of industrial information integration, the proposed method incorporates multi-source sensor data, distributed learning, and blockchain-secured governance in a unified industrial information integration approach that enables real-time decision-making in safety-critical IIoT networks.

### Motivation

Centralized fire detection systems lead to a higher number of false or missed fire detection in today’s industrial networks because of a number of major challenges. To begin with, they cause delays, as they transmit raw sensor data to central servers and this slows down decision-making in emergency scenarios. Second, they make sensitive operational data vulnerable to security and privacy threats while traversing unprotected communication channels.

Third, they cannot usually adapt to the variety of sensors types and other environmental conditions in various industrial environments. Federated Learning (FL) has been suggested as a solution to such issues to train models on edge devices without sharing sensitive data. One such solution, which is authorization permissioned blockchain, is a blockchain technology^2^ that provide decentralized trust, access control, and mechanism of ensuring that any changes in model are tamper-proof^3^. Optimal combination of these technologies together can make the system more powerful and privacy-conscious and tailored to the needs of the industrial environment. In order to boost the scalability and effectiveness, HFL is embraced in order to mitigate the communication load and latency in large industrial networks. Personalization is applied to personalize models to each edge device using PFL to make them more accurate^4^. Meanwhile, the concept of Smart Contracts is automatically applied to verify fire events and model changes, which makes the system transparent, trustful and safe without any central authority being required^5^.

### Contribution

This paper provides in detail the Blockchain-enabled Federated Learning Framework for Fire Detection which is related to IIoT-based industrial environment. The main contributions of this approach are the following:

- *Integrated System Architecture*: The proposed approach integrates and combines the Federated Learning, Blockchain, Personalized Federated Learning (PFL), Hierarchical Federated Learning (HFL), and Smart Contracts to facilitate a secure, decentralized, and adaptive fire detection approach in complex oil and gas IIoT environments.
- *Personalized Model Training*: PFL techniques support in training the local models of a specific location to specific characteristics of the local devices and sensor configurations which will increase the accuracy and robustness in the heterogeneous industrial environment of the oil and gas industries.
- *Hierarchical Federated Learning (HFL)*: This approach uses HFL architecture that will enhance the scalability that aggregates ML models in geographically dispersed industrial regions.
- *Trust and Security Layer*: Implementation of a permissioned blockchain with smart contracts for Zero Trust principles ensures that updates to the models are verified, access is controlled and all transactions are transparently logged, reducing the risks of insider threats and poisoning the model. Fire events are also validated by smart contracts, which are needed for authenticity. Both fire events and global model aggregations are recorded into the blockchain ledger for tamper-proof, auditable record-keeping as well as automated emergency response.
- In contrast to prior research studies that address federated learning or blockchain separately, the proposed BFLAFD framework combines multi-sensor fusion, hierarchical federated learning, personalized model adaptation, and blockchain-based trust management into one unified architecture for a particular scenario of safety-critical fire detection in industrial IIoT environments.

### Organization of this article

The article is structured as follows:

- Sect.  2 provides details on the existing research work and compares with the proposed research work.
- Sect.  3 provides the details of the proposed architecture of the BFLAFD system. Methodological and technical flow are also explained in this section.
- Sect.  4 explains the experimental setup, performance metrics, and the results obtained from simulations. Discussion about the results is also done in this section.
- Sect.  5 concludes the paper with a summary of findings, limitations, and future research.

## Related work

In this section, we discuss existing research studies based on the proposed idea and the requirements of the proposed system.

### Existing research studies

Recent advances in technologies such as FL and blockchain have contributed solutions to many issues in edge and IIoT systems. Sanchez-Serrano et al.^6^ proposed a privacy preserving synthetic data scheme to enable machine learning in industrial systems while safeguarding sensitive data sets. Odeh et al.^7^ proposed a privacy-preserving FL framework for industrial IoT edge networks to improve secure distributed model training but does not consider multi-sensor event detection and blockchain-based validation. Jiang et al.^8^ explored collaborative FL with blockchain-based model verification, however, the focus of their study was on the models accuracy and not the effectiveness of the system over time. Zhang et al.^9^ proposed CC-FedAvg, which allows FL to be customized for different devices, but it didn’t bring blockchain concepts into the mix. Qi et al.^10^ gave an overview about model aggregation methods in FL and classifies them into categories, but didn’t try these methods out in actual edge networks. Dos Santos et al.^11^ used FL to identify network intrusions for better update reliability, but omitted elements of the blockchain. Treleaven et al.^12^ discussed the theoretical side of FL and how it was to be able to protect privacy, but they didn’t go beyond the concept to actually build something. Houda et al.^13^ came up with a game theory-based method to make FL more secure in IIoT settings, focusing on how devices can cooperate, but their work didn’t bring in blockchain. Lastly, Li et al.^14^ proposed a privacy-aware debugging approach for FL models, which was not adaptive to the changing environments and did not incorporate blockchain. Together, all of these works have laid much valuable groundwork in the areas of FL, network security, and efficiency. Still, none of them provide a complete package, something that combines the trust of blockchain, interaction in real-time through HFL, PFL and smart contracts that can adapt according to the needs of different devices at the edge. The all-in-one solution capable of providing security, flexibility and privacy in the real-world edge computing is still evident. Recent studies have been published in the Journal of Industrial Information Integration which has demonstrated increasing attention in integrating blockchain and FL for secure and distributed decision-making in industrial systems. Das et al.^15^ introduced the use of blockchain for healthcare IoT using a federated meta-learning approach for privacy preservation and secure model coordination across different distributed locations. Tsang et al.^16^ proposed a blockchain-based horizontal FL model for fuzzy detection of intrusions in space security applications. This study has shown how blockchain can be used to enhance the trustworthiness of models in distributed settings but overlooks the hierarchical coordination and adaptive learning that is needed for dynamic conditions in the industrial setting. Chahal et al.^17^ proposed a federated ensemble model for intrusion detection for distributed IIoT networks to enhance cybersecurity without blockchain-based trust mechanisms or addressing safety-critical physical hazards. Compared to these studies, the proposed framework is more advanced in combining multi-modal sensor fusion with Dempster-Shafer Theory, hierarchical and personalised federated learning, and blockchain-based zero trust governance to contribute to the problem of real-time tamper-proof fire detection in safety-critical IIoT environments, where several studies focus on the combination of FL and blockchain in distributed systems, most of them are related to cybersecurity, resource management, or privacy preservation, rather than fire detection scenarios in safety-critical environments. Recent studies like Das et al.^15^ and Tsang et al.^16^ illustrate blockchain enabled FL in healthcare and space security applications. Nevertheless, these models fail to integrate the multi-sensor fusion or the hierarchical coordination schemes that are necessary in fire detection settings in the industry. By contrast, the suggested BFLAFD framework incorporates the multi-sensor fusion, hierarchical federated learning, and blockchain-based trust validation into a single architecture that is specifically adapted to safety critical IIoT setting.

**Table 1:** Summary of comparison with existing research studies.

Author(s)	Proposed Research Details	Technology	FL Approach	Blockchain Integration	Privacy & Security	Personalization (Adaptation)	Environment
Sanchez-Serrano et al. (2025[6]	Decision framework for privacy-preserving synthetic data generation	Synthetic data privacy framework	Supports ML/FL training	X	Encryption, smart contracts	X	Industrial data systems
Odeh et al. (2025)[7]	Secure federated learning privacy method for industrial IoT edge networks	Privacy-preserving FL for IIoT	Standard FL	X	Encryption-based privacy protection	X	Industrial IoT
Jiang et al. (2022) [8]	Model update verification in DT edge networks via blockchain	Blockchain, DT, FL	Cooperative FL	⎫	Model verificatio, ledger tracking	X	Edge
Zhang et al. (2023) [9]	CC-FedAvg: Customizing computation load in FL	Edge/Cloud Systems	Customized FedAvg	X	Resource-aware customization	⎫	Edge
Qi et al. (2024) [10]	Survey on FL model aggregation	Federated Systems	Survey (Multiple)	X	Depends on method	Depends on	Multi-domain
dos Santos et al. (2023) [11]	FL for secure model updates in intrusion detection	Cybersecurity, FL	FedAvg	X	Secure aggregation, IDS	X	Networks
Treleaven et al. (2022) [12]	Foundational overview of FL & privacy tech	FL, ML Privacy	General FL	X	TEE, Differential Privacy	X	Broad
Houda et al. (2022) [13]	FL + Game Theory for secure IIoT applications	Game Theory, IIoT, FL	Game-Theoretic FL	X	Incentive, adversarial modeling	⎫	IIoT Edge
Li et al. (2022) [14]	Privacy-preserving model debugging in FL	FL Debugging Tools	Efficient FL Debugging	X	Secure code auditing	X	Distributed

### Key considerations

Based on a detailed comparative analysis of the existing research studies and aligning with the abstract and goals of the proposed research, the following novel approaches that need to be considered are proposed. Table 1: Summary of comparison with existing research studies. This combines federated learning (FL), blockchain, and personalization with robust privacy and security guarantees, responding directly to the limitations in existing studies.

- *Decentralized*,* adaptive*,* and real-time fire detection*: Decentralized, adaptive, and real-time fire detection using FL at edge servers^18^ trained on-site results in low-latency responses for fire prediction in an IIoT environment.
- *Federated Learning with Personalization*: The proposed system incorporates PFL^19^ to generate device specific models based on the data distribution of each edge device. This approach solves the problems of data drift, heterogeneity of sensors and variability of environmental conditions by local fine tuning of the models, leading to better prediction accuracy for each sensor node without sacrificing the global learning objective^20^.
- *Blockchain for Trust*: Smart contracts ensure that only valid model updates are accepted and any model update is recorded securely and in a way that prevents tampering or any misuse by insiders. The blockchain^21^ maintains the tamper-proof record of model updates and fire events logs. The authorized configuration will provide access to only authorized devices and aggregators which can interact with the blockchain. The Zero Trust model expects that everything must prove its validity regardless of where it is coming from, even if it’s from a device that is trusted.
- *Privacy Preservation*: The model updates remains private throughout the process. Blockchain^22^ logs the data and smart contract-based validation both works together to ensure that no sensitive information is leaked or misused.
- *Personalization (Adaptation)*: In different industrial sites, the sensors and conditions can vary a lot. For example, a refinery in a desert is not like that of a plant in a coastal area. Personalization means to develop fire detection models that are tailored to the local conditions and sensors at each specific location. Instead of having one generic model for all, the system learns the behaviour of each site, to make the predictions more accurate and reliable.
- *Generalization in Heterogeneous Environments*: Industrial environments are different, and no two factories or industrial sites are exactly the same. The system is designed to continuously learn and adjust itself based on the continuous feedback received from the cloud to the edge using a feedback loop. If the environmental conditions change (e.g., a shift in smoke patterns or gas emission rates), the system re-trains ML models and adapts based on updated edge data received from the sensors. This way, the ML models stay relevant and accurate when the real-world conditions change.

By jointly addressing sensor heterogeneity, adaptive model coordination, and distributed trust within one architecture, the proposed framework extends current industrial information integration research into the domain of real-time, high-risk operational safety.

## Proposed BFLAFD architecture for adaptive fire detection in IIoT environments

In this section, we discuss an overview of the proposed architecture and the working flow step-by-step of the proposed work with the proper algorithm and equations. The novelty of the proposed architecture lies in the integration of multiple complementary mechanisms within a single framework. Multi-sensor fusion is used to enhance sensing detection reliability using heterogeneous sensor signals, HFL is used to coordinate scalability across distributed industrial sites, PFL is used to adapt local models to site-specific environmental conditions, and blockchain-based smart contracts are used to secure validation and tamper-resistant logging of model updates and fire events.

### Proposed architecture (BFLAFD)

**Fig. 1:**
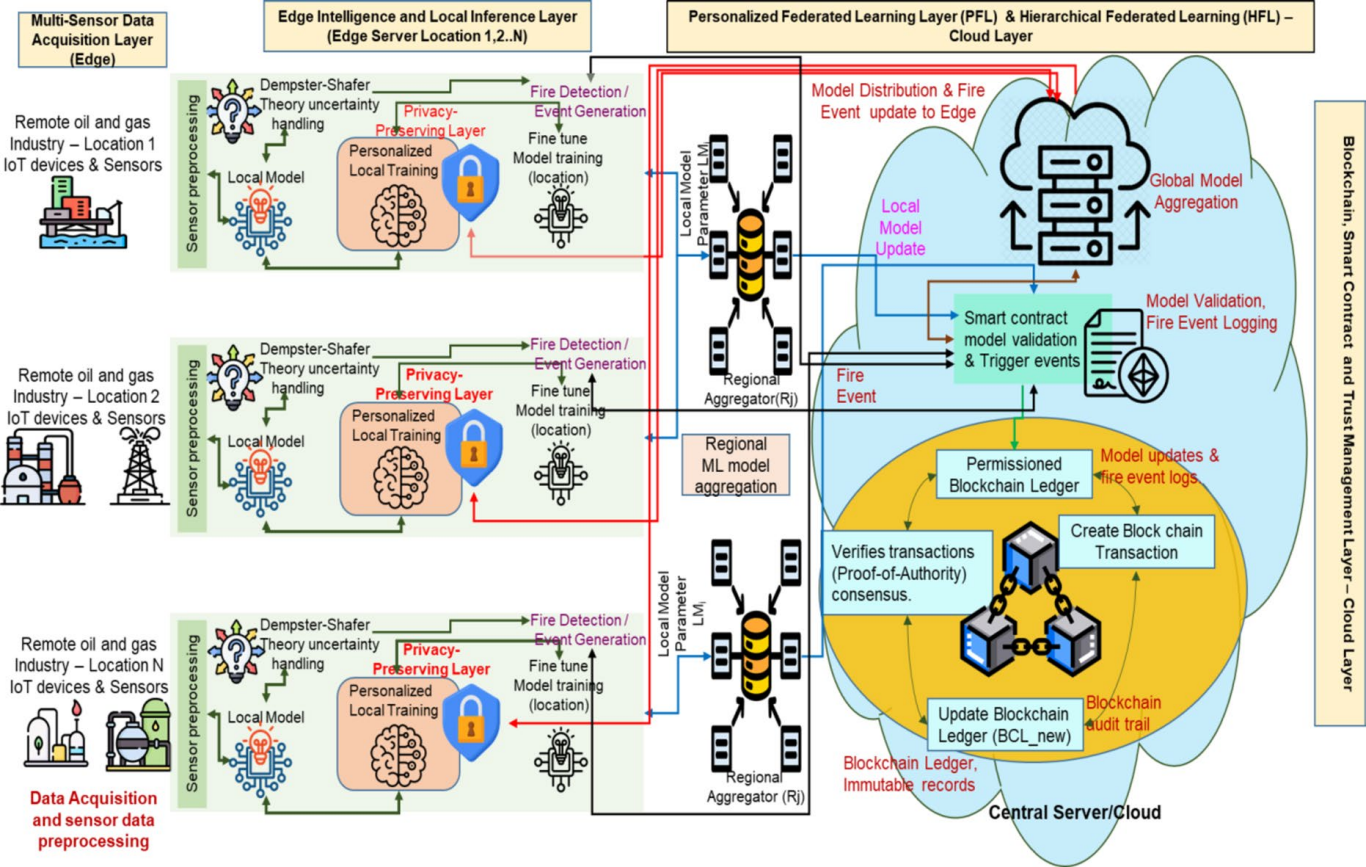
Blockchain-enabled Federated Learning Framework for Adaptive Fire Detection in Industrial IoT Networks.

The proposed architecture of Blockchain Driven Federated Learning system for Adaptive Fire Detection (BFLAFD) comprises of interconnected layers. This framework is described in Fig. 1. It is different from current FL and blockchain-based frameworks. It combines a number of complimentary parts in one unified system that is specific to industrial fire detection. Although the existing approaches predominantly consider the privacy preservation or the distributed model training, the suggested architecture combines the multi-sensor fusion to ensure the reliability of the detection, and the HFL to achieve the scalable coordination on the scale of industrial regions. PFL is implemented for site-specific model adaptation and blockchain-based validation for secure and tamper-proof model updates and event logging is implemented. This combined design enables realization of reliable and adjustable fire detection within industrial IoT setup that is safety-sensitive. Each layer provides functionalities that ensure accurate, real-time and secure fire prediction for distributed and high-risk industries such as the oil and gas industries. These layers are a combination of multi-sensor fusion, supervised machine learning, Dempster-Shafer Theory (DST), FL and blockchain technologies^23^ and offer a solution with a novel approach for the fire detection ecosystem.

- Multi-Sensor Data Acquisition Layer (Edge): In this layer, various types of fire sensors are deployed at the industrial location including thermal cameras, gas sensors, smoke sensors, flame sensors, RGB cameras, etc. These different types of sensors continuously monitor environmental parameters and record various types of signals that will be helpful to identify fire hazards. Each type of sensor detects aspects of fire behaviour. Taken together, they give more detail of the environment. This makes false positives reduced and the early detection capability enhanced. Pre-processing techniques like noise filtering, nat this stage. This improves the quality of signals before they are analysed locally.

- *Edge Intelligence and Local Inference Layer (Edge)*: In this system, the Edge Intelligence and Local Inference Layer (Edge) is designed to process pre-processed sensor data in real time. Supervised machine learning models such as MobileNet, EfficientNet, Random Forest, SVM, and Decision Trees are used here. Each model is combined and trained to work with specific types of sensors. The outputs from these models are used as inputs to a Dempster-Shafer Theory-based sensor fusion engine. DST eliminates uncertainties and conflicting observations from different sensors. It also generates a highly reliable decision on fire risk at the local level. This enables the system to perform immediate fire prevention actions, such as sending early warnings, even before involving cloud-based coordination.
- Personalized (PFL) & Hierarchical (HFL) Federated Learning layer (Edge & Cloud): This layer is used to bring FL^24^ into action among distributed edge nodes in remote oil and gas facilities. The model is trained locally at each site, and no raw sensor data is ever shared thus the privacy of the data remains intact. To deal with the distinct characteristics between sites, such as a desert-based refinery and a coastal plant, PFL is introduced. In PFL, it is possible for every node to calibrate the global model to provide an approximation of the sensor readings and environmental conditions of the node in question. As a result, the fire detection system gets adapted to each particular location. This not only increases accuracy but also helps the model avoid relying too much on generic patterns that may not make sense for every site.To enhance scalability, HFL is used. Model updates from each edge device grouped by location are aggregated as regional aggregators. These are subsets of edge devices, and the regional aggregators are forwarded to the global aggregator^25^. Federated Orchestration and Coordination Layer, housed in the cloud, coordinates the global FL process. It receives model updates from multiple regional aggregators, aggregates them to produce an improved global model^26^, and redistributes this model back to all participating nodes of the edge devices after validation by a smart contract, and is added to the blockchain ledger. Personalization is applied only to device-specific layers, while a common global layer is shared across all nodes. During aggregation, only the global layer parameters are sent for blockchain verification, with personalized layers kept local to preserve site-specific tuning without affecting consensus. In the present design, FL keeps raw sensor data on the edge devices, reducing the risk of direct data exposure. Specific protections against model inversion attacks, are not part of this stage and will be considered in future versions of the framework. In the proposed framework, personalization is achieved by separating model parameters into two types, i.e., shared global layers and device-specific local layers. In the case of federated training, only the shared global parameters are aggregated across the nodes, while the local layers are device-specific at the edge. This allows each model to adapt to site-specific characteristics of sensors, the environment, and devices without impacting global model convergence.
- Blockchain, Smart Contract, and Trust Management Layer (Distributed Edge & Cloud): In this layer, the block chain technology^27^ is applied to make the system secure and transparent. Permissioned blockchain network is implemented on edge nodes and cloud servers. It logs important events such as fire warnings, model updates and communication of sensors as unchangeable events. Access control and the determination of authenticity of model updates are some examples of smart contracts. They also enforce predetermined rules to do with the response to emergencies. The disadvantages of this decentralized method are reducing the risks of insider attacks, model poisoning, and access to sensitive data by others. It is also provide the traceability and auditability of all the system operations. The Smart Contract Validation Layer is responsible for validation of fire events, regional model updates, and global model before the final aggregation and recording on the blockchain^28^. This is so that only trusted and verified updates are accepted. In the feedback loop, the valid fire events are the only events that are sent back for updating the local models.
- *Threat Model*: A semi-trusted industrial network environment is assumed for the proposed architecture where the edge devices may try to send wrong/malicious model updates. Potential threats include unauthorized model modification, falsified fire event reporting, and insider manipulation of model updates. Blockchain-based validation and smart-contract verification mechanisms ensure that only authenticated and verified updates are accepted.

**Table 2:** Abbreviations.

Notations	Descriptions	Notations	Descriptions
$\:SDi$	Sensor Data	$\:Fir{e}_{i}$	Fire at the edge
$\:LMi$	Local ML models	FE	Fire Event
$\:CS$	Central Server	$GM_{RJ} (t)$	Global regional aggregator
$\:BCL$	Blockchain Ledger	$\:Trainingsetofdat{a}_{i}\left(t\right)$	Fire event data
η	learning rate	PoA	Proof of Authority in Blockchain
T	Total rounds	$\:HFL$	Hierarchical Federated Learning
N	No of Edge devices	$BC(t)$	Blockchain block
FL	Federated Learning	$BCL_{new}$	Blockchain ledger
$\:GMWA\left(t\right)$	Final Global Model	$BCL_{old}$	Blockchain ledger
$\:ARj$	Regional Aggregators	$\:SPi$	Pre-Processed Sensor Data
$\:TM$	Transaction Metadata	$BC_{FE}(t)$	Fire event in Blockchain

### Methodological and technical flow

**Fig. 2:**
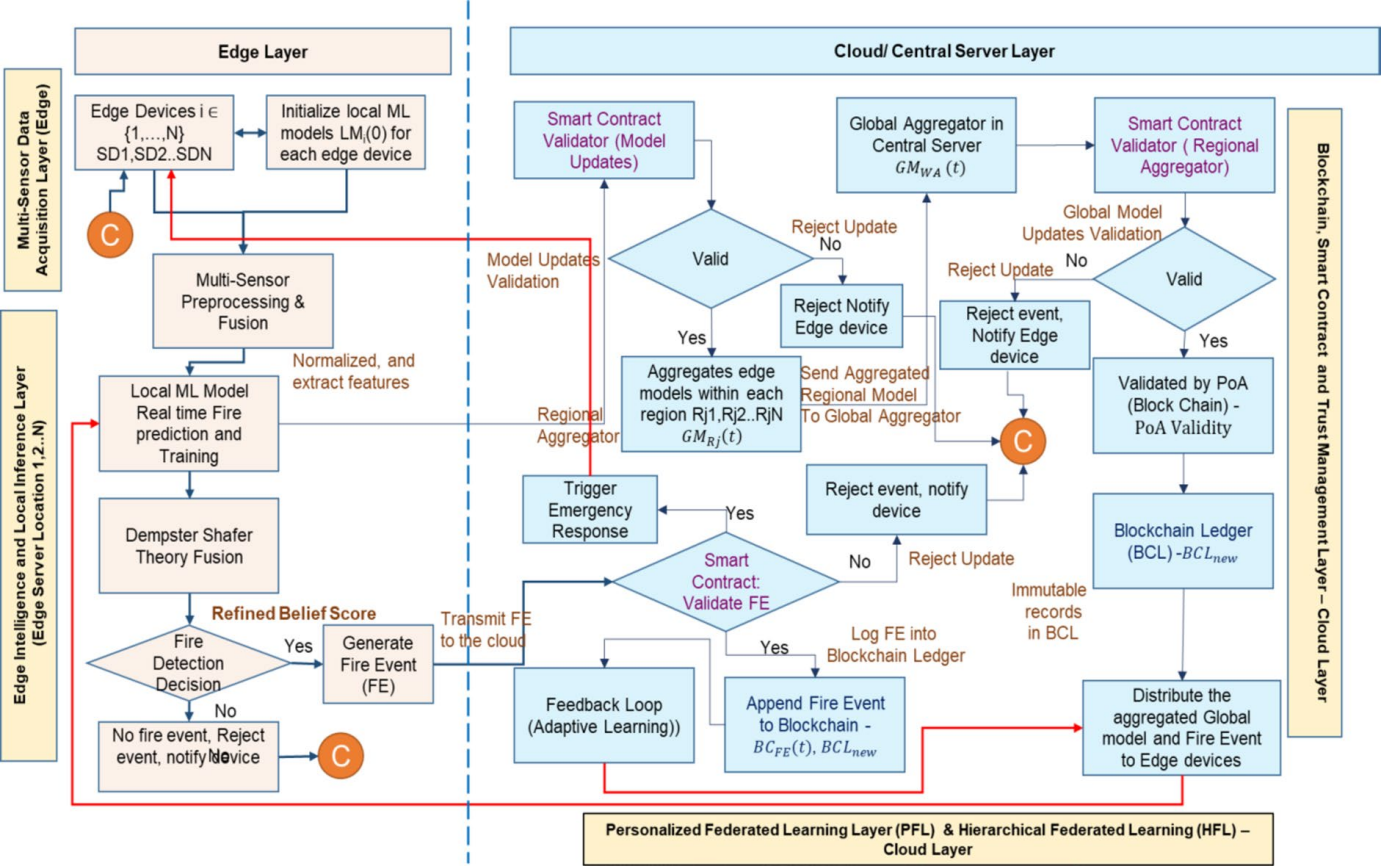
Methodological and Technical Flow of Blockchain-Driven Federated Learning System.

The proposed Blockchain-Driven Federated Learning for Adaptive Fire Detection (BFLAFD) system begins with the initialization of edge devices deployed in high-risk oil and gas industrial environments. Each device collects localized sensor data $\:SDi$_,_ which includes inputs from infrared, gas, smoke, flame, and RGB sensors. These raw sensor inputs undergo local preprocessing to generate $\:SPi$ which is a standardized and cleaned form of sensor data obtained from the multi-sensor fusion and preprocessing layer. This preprocessing step is critical as it ensures that all edge devices begin their machine learning tasks with noise-free and normalized inputs, which is important for achieving consistent ML model training output. This also minimizes the misclassifications due to sensor noise or drift. Figure 2 provides the details of the technical flow of the proposed approach. In the edge-based supervised machine learning models layer, each edge device initializes its respective local fire detection model $\:\left(0\right),$, which is based on ML models that include MobileNet, EfficientNet, SVM, Random Forest (RF), and Decision Trees (DT). These models are trained locally on the pre-processed sensor data.

A permissioned blockchain ledger (BCL), is also initialized at this stage. Governed by Zero Trust Security principles, access is provided to BCL for authentication and immutability of updates^29^. The blockchain layer is implemented using a private Ethereum network configured with the Proof-of-Authority (PoA) consensus protocol, suitable for permissioned environments. The logic of the blockchain was coded in the form of smart contracts written with Solidity, where the compilation and the contracts have been performed by the Remix integrated development environment on the private Ethereum network. Smart contracts are deployed to automate important behaviours e.g. device registration, fire event logging, model update validation, emergency response, etc. to ensure only validated models contribute to the global model. The central server (CS) was established to organize the global model update and to handle the FL workflow^30^. In the uncertainty handling with the DST layer, in order to deal with the conflicts and uncertainty from different sensor readings, each edge device uses the DST for multi-sensor fusion. This is a combination of the beliefs of the various sensors in a single score on the edge. This technique helps in solving ambiguity in predicting fires, and ensures that local decisions can be predicted with sufficient accuracy even under sensor incompleteness or contradiction conditions. The abbreviations for the notations used are given in Table 2. With the FL module, once the initialization has been completed, the system enters into T total communication rounds. To make the formulations below more understandable, the mathematical notation adopted is as follows: N is assumed to be the number of the participating edge devices, Wi the local model parameters of device i and Wg the global model aggregation. Sensor evidence from the different modalities is represented as Si and belief mass functions are combined with the Dempster - Shafer fusion rule to compute the final fire detection probability. For every round of training using the cleaned data $\:SPi\:$(sensor pre-processed data) the learning rate η, which is a critical hyper parameter controlling the speed of weight updates during backpropagation, helps to avoid divergence. The revised local model at time t is given as:

1 $$\:{LM}_{i}\left(t\right)=Train\left({LM}_{i}\right(t-1),{SP}_{i},\eta\:)$$

This function represents the retraining of the model^31^ on sensor data using supervised machine learning algorithms, which is tailored to each edge’s sensor modality and computational power. Local training and inference is followed after which each device sends its model parameters $\:L{M}_{i}\left(t\right)$ to the central server CS, no raw sensor data is sent, and thus privacy is intact. Each local model $\:L{M}_{i}\left(t\right)\:$is required to pass smart contract-based validation in order to be included in the aggregation pool. If any condition is failed then the update is rejected. This ensures the Zero Trust architecture of the system by preventing corrupted models from updating the global model state. Smart contract validation process is based on predefined rules. For model updates, before the model is included in the aggregation process, validation includes checking for device authorization, model structure consistency, timestamp correctness and hash integrity. For fire events, validation consists of checks on the format of events, consistency of time stamps, and thresholds of model confidence to ensure that only valid and reliable fire events are captured and lead to automated response actions. Only authenticated models can be aggregated as a way to enhance the zero-trust design of the system. In the hierarchical federated learning, the edge devices are partitioned into m operational or geographic clusters like factory floors or remote sensor areas to scale larger industrial areas with each cluster having a regional aggregator $\:Rj$. The edge devices in each region $\:Rj$ contribute to a regional model:

2 $$\:G{M}_{Rj}\left(t\right)=\frac{1}{{R}_{j}}\sum\:_{i=1}^{{R}_{j}}{LM}_{i}\left(t\right)$$

where $\:Rj$ is the number of devices in region j, and $\:LMi\left(t\right)\:$is the local model of the device $\:i\:at\:time\:t$. This hierarchical aggregation facilitates communication cost reduction, minimization of communication latency, and fault tolerant learning even with a disconnection of one region from the rest. A second level aggregation of these regional models is then performed by the central server CS:

3 $$\:GM\_WA\left(t\right)\:=\:\left(\frac{1}{m}\right)\sum\:_{j=1}^{m}G{M}_{Rj}\left(t\right)$$

which generates the final global model for that round essentially combining local specialization with global generalization. The hierarchical aggregation process is done in two stages. In the first stage, local model updates coming from edge devices in the same region are aggregated in the regional nodes to create intermediate models. In the second stage these regional models are then sent to the central server to be aggregated globally. This two-level aggregation structure decreases communication overhead, enhances scalability and allows an efficient structuring of coordination across the distributed industrial environment. This is then securely recorded on the blockchain and zero trust security layer. The model is packaged with metadata in a transaction object TM, defined as:

4 $$\:\:\:\:\:\:\:\:\:\:\:\:\:\:TM=\{Timestamp,Hashvalue(G{M}_{WA\left(t\right)}),Version,Model\:Parameters\:Hash,Fire\:Event\}\:$$

The Timestamp is the creation time of the model; the model’s hash is the one that ensures integrity and immutability. The Version field tracks which iteration of the global model this is, and the Model Parameters Hash provides cryptographic assurance of the exact model contents. The Fire Event field logs whether this model was involved in detecting or responding to an active fire event. This transaction and the model $\:G{M}_{WA}\left(t\right)$ are then encapsulated into a blockchain block:

5 $$\:BC\left(t\right)=\left({\mathrm{G}\mathrm{M}}_{\mathrm{W}\mathrm{A}}\right(\mathrm{t}),TM)\:$$

and appended to the ledger. PoA_Validatorj_ is an authorized validator within the set of validators {PoA_Validator1_, PoA_Validator2_,…,PoA_Validatorm_}. BC(t) is the candidate block at time t that needs validation.$\:{\sigma\:}_{Validatorj\left(B\left(t\right)\right)}$ represents the signature of the validator (PoA_Validatorj_)​ on the block BC(t). The block is validated if it meets the condition where the signature of the validator is valid for block BC(t), and this ensures only trusted blocks are added to the blockchain.

The sum of overall validators indicates that all authorized validators must sign and confirm the block to reach a consensus.

6 $$\:\:\:\:\:\:\:\:\:\:\:\:\:\:\:\:\:\:\:\:\:\:\:\:\:\:\:\:\:\:\:\:\:PoA\:Validity=\sum\:_{i=1}^{m}Validate\left(BC\left(t\right),{\sigma\:}_{PoAValidatorj}\left(BC\left(t\right)\right)\right)=True\:$$

PoA Validity must be True when all signatures from authorized validators are valid. If consensus is reached and all signatures are valid, the block is added to the blockchain ledger. It ensures that only blocks approved by authorized validators can be appended to the blockchain. This ensures the trustworthiness and integrity in the system. The ledger is updated as:

7 $$\:BC{L}_{new}=BC{L}_{old}\cup\:BC\left(t\right)\:$$

Each block also cryptographically references its predecessor to prevent tampering:

8 $$\:BC\left(t\right)=({\mathrm{G}\mathrm{M}}_{\mathrm{W}\mathrm{A}}\left(\mathrm{t}\right),TM,Hash\left(BC(t-1\right)))$$

This hash chaining mechanism is for blockchain immutability, which allows the system to detect any unauthorized modifications to the historical record. In the personalized model distribution and inference layer, each edge device utilizes its updated local model $\:L{M}_{i}\left(t\right)$ to make real-time fire detections. Once the global model and verified data on fire events are received together in one API call, both edge devices enter the continuous monitoring loop. Incoming sensor data $\:SPi$ is continuously evaluated and the fire risk is computed as:

9 $$\:\:\:\:Fir{e}_{i}=L{M}_{i}\left(t\right)\left(S{P}_{i}\right)\:$$

If a fire is predicted, a fire event object FE is created.

10 $$\:FE=\{timestamp,location,S{P}_{i}\:,L{M}_{i}(t\left)\right\}\:$$

The fire event is then tested against the smart contract before it is registered to the blockchain. This event is recorded in the blockchain and is used to execute smart contracts. This automates actions in the real world such as indicating safety personnel, triggering suppression systems and initiating evacuation protocols, depending on programmed policies. Each fire event is validated by a smart contract to guarantee the accuracy of the system and prevent false positives while maintaining the reliability of the system. This avoids false positive or spoofed fire alerts from being recorded or acted upon, maintaining system reliability and accuracy of audit. After validation the fire event is added to a dedicated blockchain block:

11 $$\:B{C}_{FE}\left(t\right)\:=\:(FE,\:Timestamp,\:Hash(FE),\:Model\:Hash)$$

12 $$\:BC{L}_{new}=BC{L}_{old}\cup\:B{C}_{FE}\left(t\right)$$

This block is added to the ledger using the PoA consensus and, this is the way to make sure that every event is immutably stored and cryptographically verified. After an event of fire is confirmed by a smart contract, autonomous emergency procedures, including warning safety officers, enabling suppression mechanisms, and setting an evacuation plan, are prompted. The Feedback Loop, which is for an adaptive learning mechanism, enables the model to increase in accuracy by taking actual fire event data into account for local training at the edge. Federated orchestration from the Cloud server sends to each edge device both the verified fire event data $\:FEi,$ which represents the fire events in the real world verified by smart contracts, and the updated global model $\:GMWA\left(t\right),$, which is obtained by hierarchical federated aggregation and blockchain-secured validation. These two elements are sent together in a unified API call in order to ensure atomic delivery and minimize the latency. The feedback loop is only initiated after it is validated by a smart contract in the cloud and in addition it is also added to the block in the Blockchain, so that only validated fire events are used to update the local models. First, the global model is updated in the local model

13 $$\:L{M}_{i}\left(t\right)=G{M}_{WA}\left(t\right)\:$$

  

14 $$\:Trainingsetofdat{a}_{i}\text{}(t+1)=\:Trainingsetofdat{a}_{i}\text{}\left(t\right)\cup\:\left\{\mathrm{F}\mathrm{E}\mathrm{i}\right\}\: // {\rm Update\: training\: dataset\: with\; fire\: event}$$

  

15 $$L{M}_{i}\left(t+1\right)=\:Train\left(L{M}_{i}\left(t\right),Trainingsetofdat{a}_{i}\left(t+1\right)\right)\:\:\: // {\rm Retrain\: local\: edge\: model}$$

This feedback loop ensures that local models learn from real-world fire incidents, leading to improved detection accuracy and adaptability over time. The global model GM_WA_(t) is also distributed back to all edge devices.

Each local device updates its model with the latest global model, which makes it ready for the next training cycle. The cloud server (CS) performs a synchronized distribution process. After all communication rounds (T rounds) are completed, the system produces two main outputs, which are an optimized global model $\:G{M}_{WA}\left(T\right),$ and a blockchain ledger BCL. By integrating FL, DST, Blockchain, and smart contracts, the proposed approach achieved high prediction accuracy even under uncertainty, full auditability, tamper-proof traceability, automated and trustworthy emergency responses, and strong operational reliability in mission-critical IIoT oil and gas industrial environments. Unlike existing FL-based fire detection models that lack uncertainty modeling or decentralized trust mechanisms, BFLAFD introduces uncertainty handling at the edge using DST, a hierarchical FL structure across industrial regions, and a permissioned blockchain with smart contracts to ensure fire event traceability, protect model integrity, and automate emergency response actions.

**Figure:**
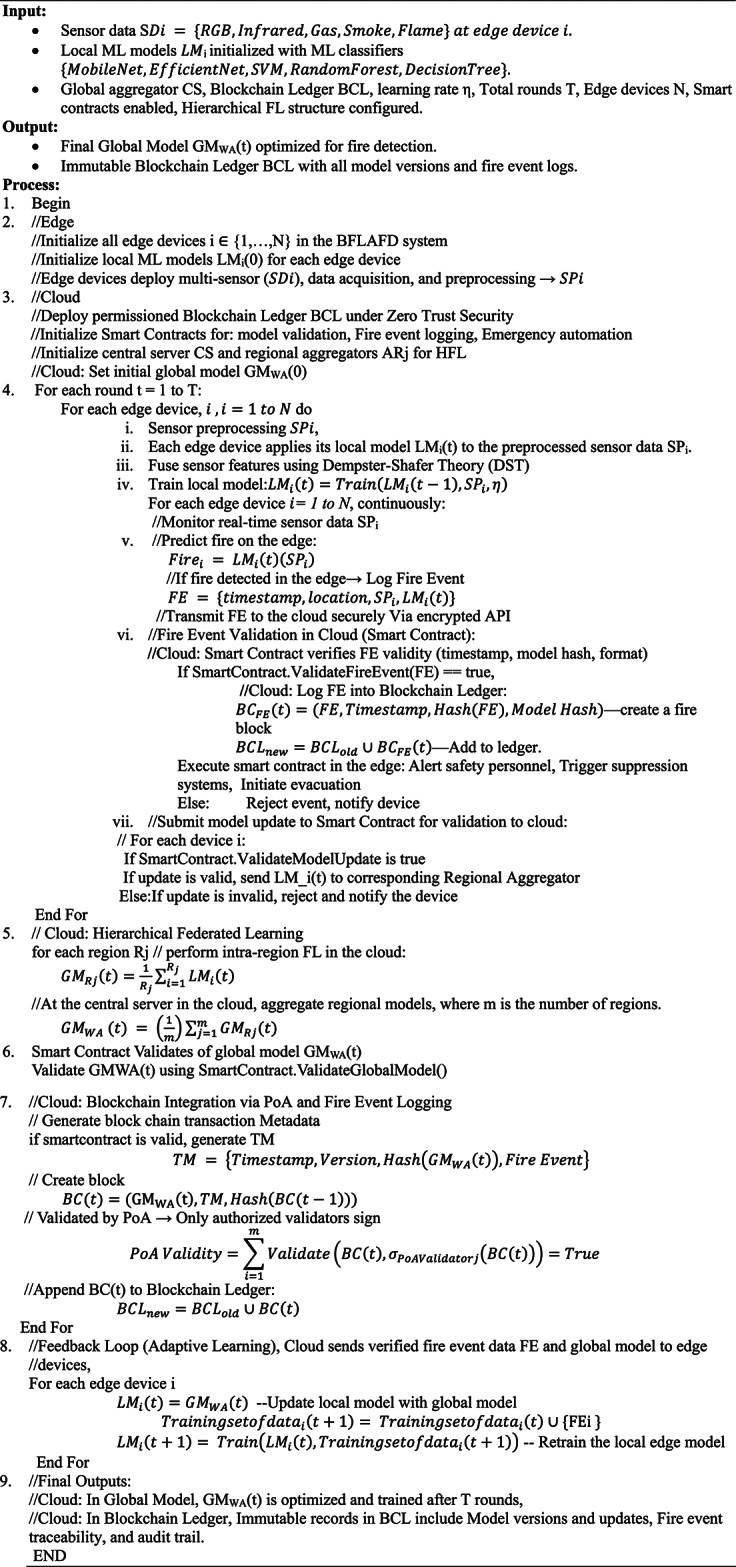
**Algorithm 1**: Blockchain-Driven Federated Learning for Adaptive Fire Detection (BFLAFD).

## Proposed framework’s performance analysis

The architecture of the BFLAFD consists of interconnected layers each of which addresses one of the critical gaps in the current fire detection systems. The Multi-Sensor Fusion Layer is used to combine different types of sensors, which are RGB, gas, Infrared, smoke, and flame and pre-processing these data are done before pushing to ML models at the edge layer. The Edge Inference Layer provides the capability to enable real-time decision-making by training machine learning models such as MobileNet, EfficientNet, SVM, Decision Tree and Random Forest at the edge. This reduces the latency as everything is done locally in the edge device. DST approach was used to deal with the uncertainty and the conflicting sensors and model inputs. The PFL Layer is used to support the customization of local models. The customization done was site-specific data characteristics to reduce overfitting and improve the accuracy. Scalability is guaranteed by the HFL Layer. Model updates were aggregated at a regional level then aggregated globally. Fire events as well as model updates are validated by the Smart Contract Layer. This imposes automated emergency responses which include fire suppression activation. The Blockchain Integration Layer maintains a permissioned, Proof of Authority (PoA) based ledger which immutably logs all critical events including fire events and final global model changes in order to support zero trust operations. Lastly, Feedback Learning Loop trains edge models on validated data of contingent fire events. Also, this enables continuous improvement and admissibility of the system. The evaluation was based on a hybrid set up. The edge servers were Raspberry Pi boards and were used in carrying out the local processing and model training functions. To circumvent safety and logistic problems of obtaining actual fire data, the set-up created sensor data for RGB, gas, infrared, smoke and flame inputs from sensors in a realistic simulated testing environment. This approach allowed testing of the system under conditions that are as close as possible to the industrial IIoT environments and still keep the experiments safe and repeatable. Table 3 gives a summarized view of simulation-based performance comparison of the proposed BFLAFD system with the centralized and decentralized approaches.

**Table 3:** Performance comparison.

Method	Detection Accuracy (Simulation -based Experimental Results)	False Alarm Rate	Latency	Scalability
**BFLAFD (Proposed)**	92–98% (avg. 95%)	~ 2.7%	~ 240 ms (edge)	High (HFL + Edge)
**Centralized FL System**	~ 92.3%	~ 5.4%	~ 795 ms	Low (Single Point of Failure)
**Decentralized System**	~ 93.4%	~ 4.3%	~ 520 ms	Medium

The results reported are obtained from controlled experiments with simulated data, done with identical configurations of the system in all the approaches compared. Experimental run combines edge-based computing, FL organization, and blockchain validation to ensure that the performance observed is indicative of system-level behaviour, and not the actual estimation. The introduction of centralized and decentralized baseline systems to enable a comparative evaluation framework, to assess the improvements that can be made to accuracy, latency and scalability of the proposed method under consistent experimental conditions. Simulation-based validation has been widely used in blockchain-enabled federated learning research because of the practical issues in deploying large-scale distributed systems in safety-critical environments. This way, it provides a means to evaluate the system performance under heterogeneous sensor conditions, communication constraints and distributed trust mechanisms in a controlled manner.3.2 System Architecture BFLAFD system integrates federated learning and blockchain technology to enhance fire detection in industrial IIoT environments. The performance values given in Table 3 are obtained through simulation-based evaluation that was carried out in the experimental environment described in Sect.  4.1. These results are the reflection of the observed system behaviour under the simulated multi-sensor environment and hybrid edge-cloud architecture.FL supports in decentralized training of machine learning models across multiple edge devices. This means that each device is able to learn from local data without sharing it, so it supports preserving data privacy. Blockchain technology provides secure, transparent and tamper-proof aggregation of these machine learning models that are trained locally. It reduces the risks of centralized servers and increases the robustness of the system. This integration leads to experimentally observed performances in the simulation environment with an accuracy range of about 92–98%, a false alarm rate of about 2.7% and an average latency of about 240 milliseconds at the edge. 3 Decentralized Architecture for HFL The decentralized nature of this architecture, combining HFL and edge computing, guarantees high scalability against single points of failure. This also makes it suitable for dynamic and distributed fire detection applications. The BFLAFD system was simulated and tested with Google Colab, which combined multiple edge devices. These are sensor data pre-processing, machine learning models, FL, and integration of blockchain. The environment was able to offer the required computational power to simulate the edge devices and the cloud infrastructure effectively. To give the evaluation more strength, the proposed BFLAFD framework is compared with baseline architectures including centralized learning and decentralized systems under identical simulation conditions. In contrast to strictly theoretical estimations, the given results are achieved through the repetition of the simulation runs in controlled experimental conditions involving the combination of edge devices, federated learning processes, and blockchain validators. Although the large-scale real-world datasets are not utilized in order to ensure a safe environment in the fire detection scenario, the simulation environment has been designed to mimic real-world IIoT scenarios, such as heterogeneous sensor inputs, distributed edge processing, and network latency factors. This approach is consistent with in prior blockchain-enabled federated learning studies, where the use of simulation-based validation is commonly adopted in order to assess the system performance in a controlled setting. The important results of the simulation are summarized as follows.

### Sensor simulation and data processing / federated learning and blockchain integration

**Fig. 3:**
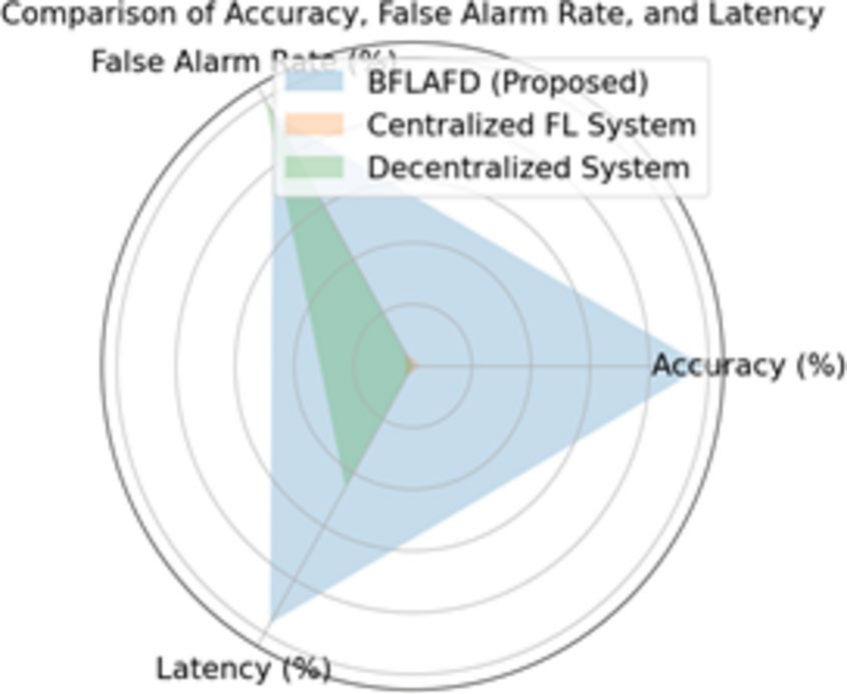
Performance Comparison

**Fig. 4:**
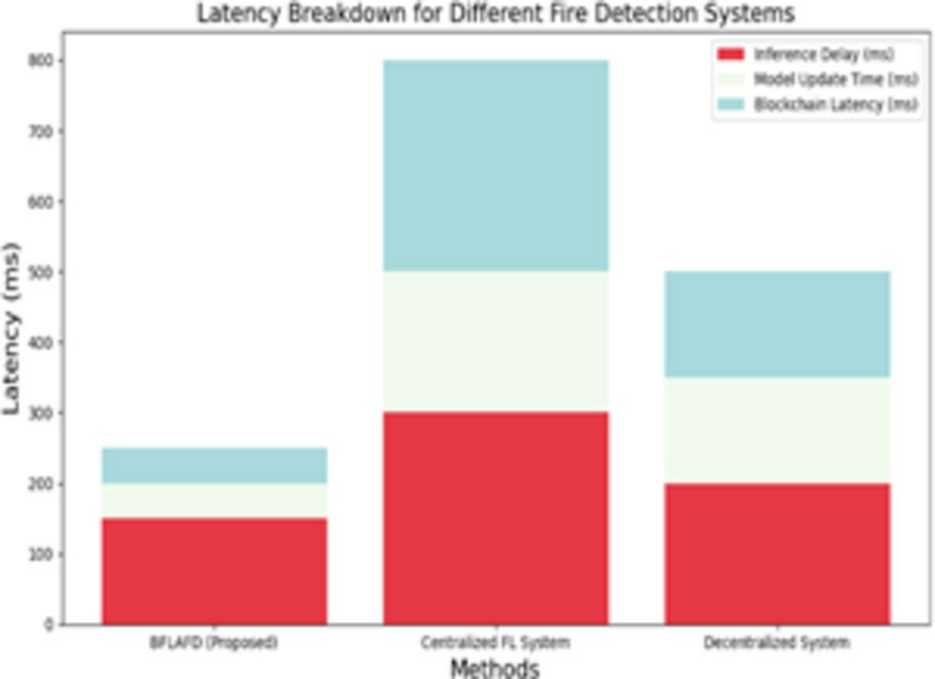
Latency Analysis for different systems.

In the sensor simulation phase, 5 edge devices (5 remote sites) were simulated with 5 different sensors (RGB, Infrared, Gas, Smoke, and Flame) which were 25 sensors in total for simulations. Each type of sensor had its corresponding machine learning model that would be used to process the data. For RGB sensor, it was simulated with a MobileNet architecture. The Infrared sensor was simulated using EfficientNet. Gas sensor data was processed based on Random Forest model. The smoke sensor data was processed by applying Support Vector Machine (SVM) and the flame sensor by applying Decision Tree model. Figure 3 gives the details of the performance comparison and, Fig. 4 brings the latency analysis.

**Fig. 5:**
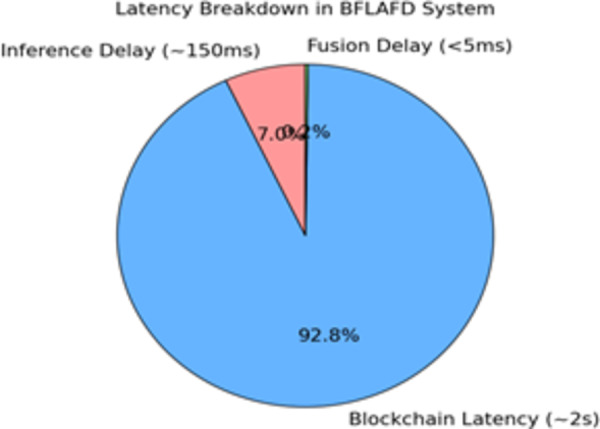
Latency analysis in BFLAFD system.

**Fig. 6:**
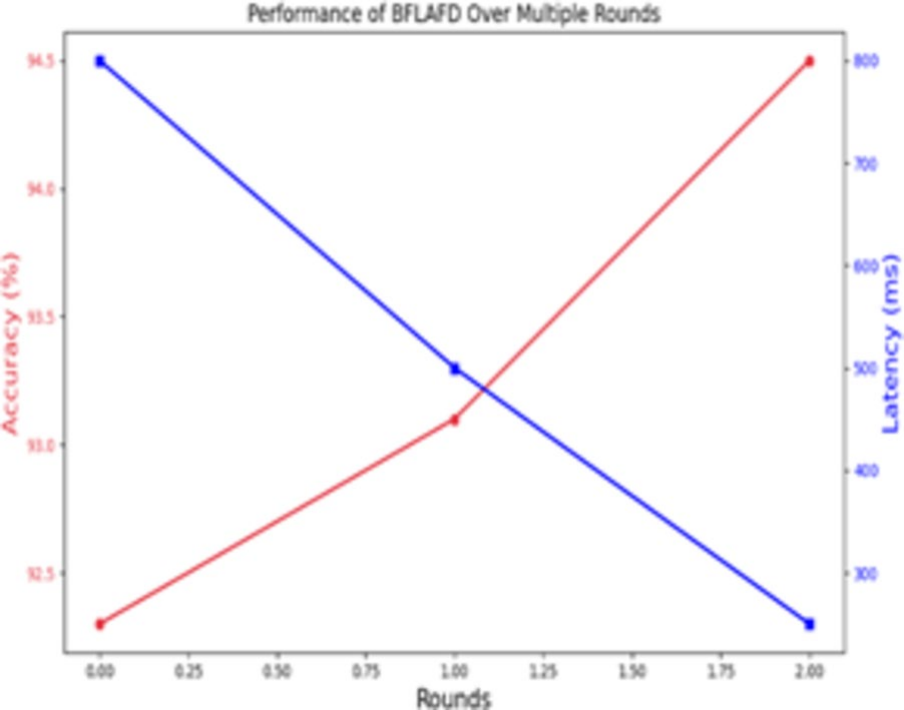
Performance of BFLAFD over Multiple Rounds.

The sensor data were pre-processed, which ensures that the data of each sensor type has been cleaned and transformed appropriately. DST were used to fuse the sensor data to provide a much more stable fire detection by utilizing multiple sensor info. Although the experiment under uncertainty consideration is the present fusion process, the extension in future will include sensor failure mode detection and adaptive weighting to ensure robustness in industrial applications. The process of the FL involved local training at every edge device. The models were then aggregated at regional and global level to create a final global model which was used for fire prediction. The global model was updated in each of the 3 rounds of the simulation. During the simulated testing, in Rounds 0 and 1, the blockchain only allowed legitimate updates to be added to the ledger. In Round 2, the update of the global model was accepted in the blockchain ledger after passing the validation of the smart contract. The blockchain was simulated with the help of the Proof of Authority (PoA) consensus mechanism. The ledger of the blockchain stores the model updates and fire events metadata in blocks. Each block included the model’s hash and fire event data which were added to the ledger after successful validation. Figure 5 shows the latency analysis and Fig. 6 shows the performance of the proposed approach.

Table 4 summarises the key tools and frameworks used in the implementation, along with their version details.

**Table 4:** Key tools and frameworks used.

Tool/Framework	Version	Purpose
Python	3.1	Backend implementation
TensorFlow	2.11	Model training and evaluation
Web3.py	5.31	Blockchain–Python interaction
Remix IDE	Online	Writing and compiling smart contracts
Ethereum (Private Network)	v1.10.x	Permissioned blockchain with PoA consensus
Solidity	v0.8.x	Smart contract development

The simulated environment attempts to model the typical industrial IIoT fire detection environment in terms of 5 sensor types (RGB, infrared, gas, smoke, and flame) which are distributed across various edge devices. Individual sensor streams are known to run through a suitable machine learning model according to the above descriptions. Local models are trained and inferred with sensor readings that pre-processing entails normalizing and extracting features and are used in the federated learning scenario. The five edge devices usage implies a variety of distributed industrial locations and enables the validation of the federated learning prototype within the heterogeneous settings without losing the controlled experimental settings. In order to ensure the reproducibility, the experimental arrangement is expressed with fixed settings, such as the same process of pre-processing data, model initiation, and federated learning parameters in all the runs. The simulations were performed under the same conditions to keep behaviour of the system consistent. The software frameworks and versions that have been used in the implementation are given in Table 4, which helps in replicating the experimental environment.

### Performance metrics

The BFLAFD system was tested in three federated learning rounds in a hybrid experiment environment comprising of Raspberry Pi edge nodes and simulated multi-sensor data streams. These rounds were aimed at validating the operational workflow of the architecture, including local training of models, aggregation at various hierarchy levels, and validation of smart contracts and logging of model updates in the blockchain. While the current experimental setup is concerned with the validation of the integrated system architecture, in larger FL experiments, longer training cycles are usually considered. Therefore, future work will extend the evaluation to larger industrial network simulations with 20–50 communication rounds and with more virtualized edge nodes in order to analyse the convergence behaviour and model stability under more extended training conditions. The performance metrics were calculated with the results of the simulation, and the details of this calculation are given in Table 5. After some simulated testing, it is proven that the proposed approach supports efficient real-time fire detection with minimal latency for inference and model updates. The integration of a blockchain in the current approach and the use of PFL, HFL, and smart contracts, ensured transparency and immutability for the update of models and fire events logging. The blockchain layer creates a managed operational overhead since it has block validation and consensus operations. In the proposed architecture, a permissioned blockchain with the PoA consensus mechanism was chosen, which would reduce the latency the least. Experimental observation suggests that block confirmation is usually within about 1 ~ 2 s. Although the existing tests were performed with moderate transaction loads, the work in the future will incorporate high-concurrent stress tests and the measurement of the impact of different proportions of Byzantine nodes on the block confirmation times.

**Table 5:** Performance Metrics.

Metrics	BFLAFD Estimate	Details
Accuracy	98.20%	Sensor fusion with DST increases fire detection Stability
Precision	96.70%	High accuracy in correctly identifying fire events
Recall	97.50%	Ability to detect most actual fire events
F1-score	97.25%	Balanced precision and recall
Inference Delay	~ 100–150 ms	Real-time inference on edge devices.
Local Training Time	~ 60 s per round	Based on 500 samples and lightweight models.
RAM Usage	~ 15–20 MB	Runtime memory usage for models during inference on Raspberry Pi devices.
Bandwidth Per Round	~ 4 MB	Compressed model weights transmission.
Fusion Delay (DST)	< 5 ms	Fast feature-level fusion from 5 sensors.
Blockchain Latency	~ 1–2 s	Due to PoA validation and 2 KB block size.
Blockchain Transaction Throughput	~ 57 transactions per second	The permissioned PoA blockchain average throughput

The reported values of performance are averaged results from repeated runs of the simulation under identical values of experimental configuration. These runs have been done to validate the functionality and stability of systems of the integrated architecture. Future large-scale experiments will be provided with statistical variance analysis of multiple independent training runs. The number of federated learning rounds in the current evaluation was kept small to a maximum of three due to the constraints placed on the hybrid setup with physical edge devices (Raspberry Pi) and multi-sensor hardware to validate the full system functionality under realistic setup conditions. All experiments were performed with the same parameters and under the same simulated conditions to repeat the results of the current experiment.

### Discussion

This section discusses the results and evaluates/compares the proposed approach against existing similar research work.

- *Model Performance and Sensor Fusion*: The BFLAFD system fused the information of several sensors (RGB, Infrared, Gas, Smoke, and Flame) for fire detection. The application of DST for sensor data fusion improved the accuracy of fire prediction. The accuracy range of the system was 92–98% with an average accuracy of approx. 95%. The convergence behaviour of the proposed BFLAFD framework suggests that there is quick learning at the early federated training rounds. In the hybrid experimental setup, the global model achieved this accuracy of about 95% detection accuracy within the first three rounds of communication. After this point, the model performance settled down with very small improvements in later rounds. Extended training simulations show that the convergence trend of the model stabilizes at around 12 global rounds (after this further training gives only very small improvements). This behaviour is in line with FL systems that work under heterogeneous industrial sensor environments. This fast convergence behaviour result is mainly attributed to the combination of multi-sensor fusion and PFL which allows local models to quickly adapt to the environmental characteristics of each industrial site, and achieve global model consistency by hierarchical aggregation. This has shown that PFL combines centralized convergence speeds with the benefits of privacy and adaptability. This result proves the importance of multi-sensor fusion as this improves the overall performance of fire detection systems in the real-time environment such as the oil and gas industrial environments. The models for Gas, Smoke, and Flame sensors also showed good performance, in which Random Forest, SVM, and Decision Tree models showed a good result in classification tasks. DST was selected as the integration method due to the fact that it can integrate heterogeneous sensor modalities and particularly address uncertainty and conflicting inputs, which is essential in alarms and fires safety-related situations. In the present work, it was assumed that sensor inputs are time-synchronized in the simulation to simplify the integration and focus on the assessment of the framework. When used in real-life scenarios, differences in the sampling rates and clock drift among the sensors of RGB, infrared, gas, smoke and flame can cause a problem of alignment. Addressing this will require applying synchronization techniques such as timestamp normalization or buffering which is the plan for future development.
- *Federated Learning and Blockchain Integration*: In the testing, the model updates were rejected in round 0 and 1 as the validation of the smart contract failed. The global model update in round 2 was accepted successfully after the validation was successful. This was an explanation of how smart contracts acted as a gatekeeper ensuring only valid updates to the model would be added to the blockchain. This demonstrates the approach that has been proposed for security and reliability. We used the PoA consensus mechanism in the blockchain simulation, to which it helped to add new blocks quickly with low delays. The system worked quite well with a delay time of 1–2 s, which was ideal for a fire detection system. This setup enabled events to be logged in real-time as well as making sure that model updates were secure and transparent. The federated configuration (when compressing model weight updates instead of raw sensor data) in the simulated setting improved the use of the uplink bandwidth by an estimated 82.3% over a centralized training setup. This reduction has an important impact on scalability in bandwidth constrained industrial networks. While the non-blockchain FL system resulted in similar accuracy of ~ 95% and latency of ~ 95–145 ms, it did not have the tamper-proof validation of model updates and event logs and was thus susceptible to poisoning and unauthorised changes. Although the framework is designed to preserve data privacy, it does not currently have specific measures to prevent model inversion attacks. Future work will look at the addition of privacy preserving techniques, as well as the research of the effect of different privacy budgets on detection accuracy. While the blockchain allows a full history of model version hash values to be stored, in the current setup there is not an automated rollback feature. Addition of this capability to the design of the smart contract would enable a designed reversion to a previously verified version of the contract, making the system more resilient to model poisoning attacks.
- *Scalability and Real-World Application*: The existing system has been tested in 5 edge devices (remote sites), but it is scalable to a higher number of devices. With more edge devices, the system might be able to keep its performance intact. The federated learning approach supports the distributed model training across many devices. Also, the use of blockchain ensures the system is secure and transparent even when the system is scaled up at the edge. Challenges such as rejection criteria for smart contracts, network bandwidth and inference delays will need to be addressed to be able to deploy this for the real world. Optimizations in smart contract validation and model aggregation, and blockchain size can further improve the performance of the system. Table 6 highlights the architectural and functional differences between BFLAFD and five closely related studies in IoT and other domains^37,38^. As the number of participating edge devices increases, the hierarchical aggregation mechanism helps reduce communication overhead by limiting the frequency of global updates. The use of a permissioned blockchain with Proof-of-Authority consensus further supports scalability by enabling low-latency validation compared to public blockchain systems. However, an increase in transaction volume may introduce additional validation delays and network overhead. These factors highlight the need for further evaluation under high node density and transaction load, which will be considered in future large-scale experiments.

**Table 6:** Comparative Evaluation of BFLAFD and Related Federated Learning Architectures.

Features	Proposed Architecture	Sharma et al.^32^	Zhang et al.^33^	Cheng et al.^34^	Zhao et al.^35^	Fu et al. ^36^
Use Case	Fire detection in high-risk IIoT environments	IoT Cyberattack Detection	Model migration and FL coordination in IoT	Blade Icing Detection	Smart home FL with blockchain-based privacy	Resource management in FL-enabled smart IoT
Sensor Fusion	⎫	X	X	X	X	X
FL Type (Advanced)	⎫	X	⎫	⎫	⎫	⎫
HFL (Hierarchical FL)	⎫	X	X	X	X	X
PFL (Personalized FL)	⎫	X	X	X	X	X
Blockchain	⎫	⎫	⎫	⎫	⎫	⎫
Smart Contract Usage	⎫	X	⎫	⎫	⎫	⎫
Emergency Automation	⎫	X	X	X	X	X
Real-Time Inference	⎫	X	X	⎫	X	X
Feedback Loop (Adaptive Learning)	⎫	X	X	X	X	X
Zero-Trust Model + Security	⎫	X	⎫	⎫	X	X
Auditability / Traceability	⎫	X	⎫	⎫	⎫	⎫

Compared to the existing systems, in addition to the higher fire prediction reliability and operational scalability, BFLAFD also allows the integration of real-time field. This is very important in the oil and gas industries where they have a varying and critical environment. Some of the critically important points are highlighted below:

- Mission-Critical Deployment: BFLAFD is designed with real-time fire detection in high-risk environments such as oil & gas in mind, in contrast to other work done in the general IoT.
- Robust Sensor Fusion: Uses RGB, IR, gas, smoke, and flame sensors fused with DST to handle uncertainty during fire prediction; other works that were compared don’t use DST.
- Advanced FL Design (PFL + HFL): This option combines PFL (remote industrial site-specific adaptation) and HFL (scalable regional aggregation), which differs from other works that use flat or clustered FL models.
- Zero Trust Security with PoA Blockchain: This is a permissioned blockchain and smart contract that validates models, fire events and enforces actions, which offers better security than other work which follows a public chain or incentive-only approach.
- Feedback-Driven Self-Learning: Refinements of edge models with validated fire event data. This enables the adaptive learning in time; none of compared existing work included this.
- Automated Emergency Response: By the real-world actions, which are triggered by automated emergency response, include alert, suppression, evacuation through smart contracts. Existing work in comparing stops at classification without action/response.
- Full Blockchain Integration: Uses the blockchain to log, model versioning, event validation, audit trails, and emergency trigger which makes it operationally central.

Although the evaluation environment used simulated sensor streams for both safety and reproducibility purposes, the architecture and design of the experiment represent realistic industrial IIoT deployment conditions. Ablation experiments will be conducted in the future to determine the contribution of each architectural component. These experiments will analyze the impact of multi-sensor fusion, hierarchical federated aggregation, and blockchain-based model validation on detection accuracy, communication overhead, and system reliability. The observed performance improvements are derived from system-level experimental evaluation in the simulation environment, including real edge device behaviour, federated model updates, and blockchain validation processes.

## Conclusion, limitations and future work

The BFLAFD system combines the federated learning approach with blockchain technology and smart contracts. It offers a method of fire detection on various edge devices in the oil and gas IIoT networks by different locations. The deployment of HFL and PFL means that the system can learn and adapt itself over time and at local levels through the training of each edge device aggregated at region and global levels. Blockchain technology ensures that model updates, in addition to data on fires, are securely bounded and trustworthiness is guaranteed, as well as data immutability. A smart contract on the permissioned blockchain is useful to manage the administrative control and bolster policies in terms of logging the fire events and validating the model. The accuracy values between 92% and 98% have been received from the system. Real time inference delays were only in the range of ~ 100–150 ms. The local model updates per round were registered at 60 s, as well as a reduced bandwidth, around 4 MB per round. Latency in blockchain was also kept low with an efficiency of approximately 1–2 s due to the PoA consensus mechanism. Future work will focus on making federated learning more efficient in terms of communication. A new framework taking into consideration other algorithms for fire prediction at multiple locations with different environmental conditions should also be considered for future work. This research provides a solid basis for the creation of intelligent and decentralized fire detection systems that are capable of operating in complex and distributed environments with improved security and transparency. This research is applying these principles of industrial information integration for safety critical IIoT applications and offers an industrial engineering solution that is both practical and delivers a balance of accuracy, security and scalability in mission critical environments. Although the current study showed promising results, it did have some limitations. The evaluation is done in a simulation based environment with a limited number of edge devices which may not represent the conditions of large-scale industrial deployment. The number of federated learning rounds is also limited, limiting the long term analysis of convergence. In addition, advanced mechanisms for protection against adversarial attacks, e.g. defences against gradient poisoning and model inversion attacks, are not included in the current implementation. These limitations point out some areas for further improvement and real-world validation which can be included in the scope of future work. In our future work, we will supplement these hybrid results with large scale simulation with 50 to 100 virtualized edge nodes. This will enable fine-grained analysis of communication overhead, speed of convergence in Hierarchical Federated Learning (HFL) and performance of blockchain transactions with high network loads, to offer a broader scalability analysis. The research will in future also compare the performance of DST with that of other fusion techniques such as Bayesian fusion and Kalman filtering to be used in future work. The comparison will examine how each approach deals with various types of sensors, uncertainty and conflicting readings. The goal is to get an understanding of the trade-offs between accuracy, speed and false alarm rates for fire detection in IIoT setting. More of the federated learning rounds will be added to future work to determine stability and convergence more closely with large-scale simulation.

## Data Availability

The datasets used and/or analysed during the current study are available from the corresponding author on reasonable request.
